# The Mirroring Dance: Synchrony and Interaction Quality of Five Adolescents and Adults on the Autism Spectrum in Dance/Movement Therapy

**DOI:** 10.3389/fpsyg.2021.717389

**Published:** 2021-10-14

**Authors:** Elizabeth Manders, Sharon Goodill, Sabine C. Koch, Ellen Giarelli, Marcia Polansky, Kathleen Fisher, Thomas Fuchs

**Affiliations:** ^1^Department of Creative Arts Therapies, Drexel University, Philadelphia, PA, United States; ^2^Department of Therapy Sciences, SRH University Heidelberg, Heidelberg, Germany; ^3^Research Institute for Creative Arts Therapies (RIArT), Alanus University of Arts and Social Science, Alfter, Germany; ^4^Doctoral Nursing Program, Drexel University, Philadelphia, PA, United States; ^5^Dornsife School of Public Health, Drexel University, Philadelphia, PA, United States; ^6^Psychiatrische Universitätsklinik, University of Heidelberg, Heidelberg, Germany

**Keywords:** autism spectrum disorders (ASD), dance/movement therapy (DMT), interactional synchrony, affective engagement, video analysis, embodied interactions

## Abstract

**Background:** Individuals on the autism spectrum are often described as having atypical social interactions. Ideally, interactional synchrony helps any interaction flow smoothly with each individual responding verbally, non-verbally, and/or emotionally within a short timeframe. Differences in interactional synchrony may impact how individuals on the autism spectrum experience social encounters.

**Method:** This mixed methods pilot study examined interactional synchrony in five cases of adolescents and adults on the autism spectrum through secondary analysis of video of the participants in movement-based mirroring tasks during dance/movement therapy. Raters described the movement and interactions of the participants while they were leading and following mirroring and engaged in open-ended free dances with a partner. Videos were also scored on measures of affective engagement, flow of the interaction, and synchrony.

**Results:** One of the most striking findings of this study was the difference between engagement in the instructions of the task and engagement with the partner: participants often followed the instructions for the mirroring tasks with little further social engagement with their partner. When participants did engage in moments of social initiation, attunement to the partner, and interactive behaviors, these did not develop into longer interactions. A paired *t*-test of the correlation coefficients for each participant showed that scores on synchrony and affective engagement were more strongly positively correlated in the less structured open-ended dance and in video clips of interactive behaviors, than in the videos of simply leading or following mirroring. Synchrony was also significantly more strongly positively correlated with the observed flow of the interaction than with observed affective engagement. With the small sample size, however, most of the correlation coefficients were not significant and should be tested on a larger sample.

**Discussion:** Interpersonal synchrony may not be sufficient to effectively support social engagement when individuals on the autism spectrum simply follow instructions to synchronize their movements. Synchrony-based interventions may therefore need to include more complex open-ended social scenarios as interactional synchrony may then be more correlated with perceived interaction quality. Therapists may also need to partner with participants to model using non-verbal social behaviors to develop interactions within mirroring tasks.

## Introduction

Autism spectrum disorders (ASD) involve difficulties with social communication (including difficulties with non-verbal communication and social-emotional reciprocity) and restricted or repetitive interests or behaviors (American Psychiatric Association, 2013). Many verbal individuals on the autism spectrum^1^ can learn specific social skills and at times recognize another’s perspective (Fitzpatrick et al., 2018). They may attend to faces for emotional or social cues (Guillon et al., 2014), use gestures (de Marchena and Eigsti, 2010), or other non-verbal behaviors (Garcia-Perez et al., 2007) at the same frequency as cognitively matched peers. Despite this similar frequency of behaviors, their social behaviors may continue to appear qualitatively different or these skills may vary dependent on the situation. For example, observers scored interviews of adolescents on the autism spectrum as significantly lower in affective engagement and flow of the interaction than interviews with adolescents with intellectual disability, while they only found a statistically significant difference in the frequency of one non-verbal behavior in one context (Garcia-Perez et al., 2007). It may be that some individuals on the autism spectrum can perform these social behaviors in simplified research tasks, when given explicit instructions, or when the gesture or behavior has a specific communicative meaning, while being less likely to perform the same social behavior in more socially complex or flexible everyday scenarios or when the action is performed primarily for a social purpose (Garcia-Perez et al., 2007; Guillon et al., 2014). A clearer description of the qualitative differences in non-verbal social behaviors, including a better understanding of which of these differences are meaningful, is needed to target these differences and help individuals navigate social interactions across settings.

### Autism From an Embodied Perspective

An embodied enactive perspective on ASD is offered here as an alternative theoretical perspective to Theory of Mind on the mechanisms underlying human social interaction (see Koch and Fischman, 2011; De Jaegher, 2013). This embodied enactive perspective proposes that individuals’ sensory-motor experiences within the social context drive their everyday understanding of, and engagement in, social interactions. Koch and Fischman (2011) offer emotional contagion as an example of this rapid sensory-motor level of understanding within the interaction. They explain that processes of emotional contagion: “cannot be explained in terms of conscious cognitive processing. They result from the flow of interaction, from our capacity to “map” expressions from other bodies directly to our bodies, to match or mirror expressions, to resonate bodily; only then do they sink into our cognitive-affective systems (p. 65)”.

Interactive qualities of engagement, such as rhythmic synchronicity and coordination of turn-taking with another person, can furthermore build upon themselves to maintain the interaction. Shared meanings can be created through these interactive patterns without necessarily considering abstract representations of the other’s perspective. This coordinated interaction may also develop in a way that neither individual anticipates. For example, when “encountering someone in a narrow corridor. Sometimes, as you meet, in order to avoid bumping into each other, you both step in front of each other a few times, each moving to the same side at the same time (De Jaegher, 2013 p. 6).” Here the actions, perceptions, and creation of shared meaning are co-occurring within the interaction. Processing delays or differences in attention may lead to differences in the timing of turn taking or other patterns of engagement in social interactions with individuals on the autism spectrum.

Many individuals on the autism spectrum experience under- or over-responsivity in one or more sensory modalities. Intensified, delayed, or diminished sensory experiences may make it difficult for individuals to rapidly notice, preferentially attend to, or process socially relevant information, which could impact or alter their social learning and interactions (Thye et al., 2018). Motor delays and deficits may likewise alter an individual’s experience of the social environment, particularly if they already have decreased social attention. In fact, early motor delays are correlated with later communication deficits in children on the autism spectrum (West, 2018). Although motor skills vary greatly among those on the autism spectrum, there are high rates of delays or impairments in postural control, balance, gait, motor coordination, motor planning, and anticipatory control, as well as possible impairments in upper-limb movements and fine motor functioning (Fournier et al., 2010; Kaur et al., 2018). A lack of synchrony between an individual’s words and gestures may also contribute to the qualitative differences in social communication by individuals on the autism spectrum. For example, longer time lags between speech and gestures have been found to make stories appear less affectively engaged (de Marchena and Eigsti, 2010).

### Interactional Synchrony

Smooth social interactions generally follow a well-coordinated flow of attention and responsiveness to the other person. This includes moments of interactional synchrony, or interpersonal coordination, between the two (or more) individuals’ movements, gestures, speech, physiology, or emotions (Condon, 1975; Fitzpatrick et al., 2018; McNaughton and Redcay, 2020). Interactional synchrony can involve: (a) actions that occur at exactly the same time (such as dancing in unison or walking in lock-step) or (b) responsive behaviors (such as shifting one’s posture after the other person moves or a child giggling after an adult makes a face). Studies of various forms of synchrony and automatic mimicry have found these to be positively correlated with rapport, increased positive feelings toward the other person, increased cooperation, empathy and group cohesion (Fitzpatrick et al., 2018; McNaughton and Redcay, 2020). Experiencing interpersonal synchrony can also encourage increased consideration of the other’s mental state (Baimel et al., 2018).

Several studies have found differences in interactional synchrony in individuals on the autism spectrum including delayed actions (Condon, 1975; Marsh et al., 2013), decreased “low level” automatic synchrony (Marsh et al., 2013), decreased frequency and length of complex forms of coordination (Brezis et al., 2017; Romero et al., 2018), less predictable and more variable rhythms of interaction (Delaherche et al., 2013), and differences in the rhythms and patterns of how others respond or attempt to engage them (Delaherche et al., 2013). In a review of 25 recent studies of interpersonal synchrony, McNaughton and Redcay (2020) reported that studies found differences in interpersonal synchrony between individuals on the autism spectrum and neurotypical control groups in many, but not all, contexts. In the studies of movement synchrony, the individuals on the autism spectrum generally demonstrated reduced interpersonal synchrony with social partners except in tasks with fewer social demands or when synchronizing with a computer. Decreased movement synchrony during conversations was found in adults (Georgescu et al., 2020) and children (Zampella et al., 2020) on the autism spectrum and negatively correlated with ASD symptom severity (Zampella et al., 2020). Fitzpatrick et al. (2018) found that adolescents on the autism spectrum showed decreased synchrony with their parents. Within this, spontaneous synchrony (i.e., spontaneously arising when doing the same action) and intentional synchrony (i.e., when instructed to match the pace) appeared to relate to different aspects of social interaction. Engagement in spontaneous synchrony was associated with the ability to assign feelings to objects in Theory of Mind tests, while intentional synchrony was associated with measures of attention and social responsiveness (Fitzpatrick et al., 2018). If differences in interactional synchrony contribute to qualitative differences in social interactions in individuals on the autism spectrum, interventions should promote the coordination of movement and action with others in social situations (De Jaegher, 2013; Marsh et al., 2013). Dance/movement therapy (DMT) may be an appropriate intervention to address interactional synchrony as creative movement-based activities, such as mirroring, emphasize reciprocity and motor synchrony within the context of a therapeutic relationship (Koehne et al., 2016).

### Dance/Movement Therapy

Dance/movement therapy (DMT) is “the psychotherapeutic use of movement to promote emotional, cognitive, physical and social integration of individuals” (American Dance Therapy Association, 2019). Dance/movement therapists use movement-based interventions to help individuals increase their expressivity, increase their emotional coping skills, improve self-awareness, and explore new responses to challenging situations (Levy, 2005; Kestenberg Amighi et al., 2018). Rather than teaching socialization primarily as a set of social rules, dance/movement therapists take a strengths-based approach with creative processes such as moving, relating, and responding in the moment as the interaction unfolds.

Although DMT has been used with children on the autism spectrum since the 1960s (Levy, 2005), the research is limited, and there are few large studies of DMT for individuals on the autism spectrum (Takahashi et al., 2019). Techniques such as attunement and mirroring, in which a participant leads or follows another’s movements or jointly improvises movements with a partner focus on the embodied experience of interactional synchrony, social coordination, and emotional responsivity (Feniger-Schaal et al., 2018). Samaritter and Payne (2017) found that when a dance/movement therapist attuned to (sensed the emotional tone of the actions), and responsively followed, a non-verbal child’s movements, the child’s own spontaneous interactional behaviors toward the therapist increased. These non-verbal interactional behaviors appeared to follow a development sequence, and when these interactional behaviors are combined, they “contribute to a high complexity of interpersonal engagement and attunement during shared movement actions” (Samaritter and Payne, 2017, pp. 8–9). It may be useful to investigate if adults on the autism spectrum use these foundational interactional movement behaviors. Attunement is used with individuals of all ages and is closely related to mirroring, so these techniques may be useful in exploring the use of interactional movement behaviors.

Two different 10-week DMT interventions that included mirroring and other techniques to develop interpersonal synchrony in teens or adults on the autism spectrum did not find significant changes in empathy (Koehne et al., 2016; Mastrominico et al., 2018), however one found significantly increased emotion inference, synchronization, and movement reciprocity (Koehne et al., 2016). A 7-week pilot study found a small but significant improvement in body-awareness, self-other awareness, social skills, and overall well-being (Koch et al., 2015).

Dance/movement therapists use movement observation and assessment tools to describe the characteristics of an individual’s movement. Similar to vitality affects, these characteristics describe how an individual moves rather than just what the individual is doing. These can also be related to emotions, responses to the environment, and psycho-physiological development (Bartenieff and Lewis, 1980; Kestenberg Amighi et al., 2018). These tools include Laban Movement Analysis (LMA; Bartenieff and Lewis, 1980), the Kestenberg Movement Profile (KMP; Kestenberg Amighi et al., 2018) and recently, specifically for clients on the autism spectrum, the SEAM observation scale (Samaritter and Payne, 2017). The SEAM scale tracks relational movement behaviors and their change over the course of therapy. Describing the characteristics of movement during interactions through the lens of these tools may help better describe some of the poorly understood qualitative differences in interactions by individuals on the autism spectrum.

The aims of the current mixed methods pilot study were to (a) explore the relationship between interaction quality and interpersonal synchrony, (b) observe change in interaction quality or synchrony over 10 weeks of DMT, and (c) qualitatively describe any additional characteristics of the movement that appeared to be related to interaction quality.

It was hypothesized that there would be a relationship between synchrony and interaction quality. It was hypothesized that different forms of synchrony or delayed following might vary in their relationships with interaction quality, but as an exploratory study the nature of these specific relationships was not predicted. It was hypothesized that there would be an increase interaction quality and synchrony over 10 weeks of DMT. It was hypothesized that qualitative data describing the characteristics of the movements and interactions would reveal further patterns in the characteristics of participants’ movement that appeared to have a possible impact on the interaction. It was predicted that these qualitative descriptions might help explain, contradict, or add to the quantitative results, and that this could guide recommendations for further studies.

## Materials and Methods

This study consisted of a mixed methods secondary analysis of videos of five participants in DMT groups for individuals on the autism spectrum. The video data were collected during a parent study (see description below). Raters blind to the session order described the qualitative features of the movement and interactions of the individuals on the autism spectrum, and coded the videos for interpersonal movement synchrony and interaction quality (affective engagement and flow of the interaction). These scores were analyzed for change over time and correlation between synchrony and interaction quality.

### The Parent Study

The videos for this secondary analysis were from a parent study which conducted a mirroring-based DMT intervention for adolescents and adults on the autism spectrum (Mastrominico et al., 2018). This original study was part of an interdisciplinary, multi-site project entitled *Toward an Embodied Science of InterSubjectivity* (TESIS) with research from different fields conducted at 13 associated research institutes. The parent study received approval from the Medical Faculty of the University of Heidelberg, Germany ethics board (Ethikkommission) for its human subject protection procedures including informed consent. The participants gave consent for participation, with additional parental consent for minors.

#### Participants of the Parent Study

Participants of the parent study were between 14 and 65 years old, had an IQ over 70, and were previously diagnosed with ASD, autism, Asperger syndrome, or pervasive developmental disorder according to ICD 10 criteria. The parent study used participant’s reported prior diagnosis and did not require further confirmation of the diagnosis in order to meet criteria for participation in the study. Exclusion criteria for the parent study included psychosis, addiction, or severe neurological disorders with effects on mobility. The parent study assigned 73 participants to 10 weeks of DMT or wait-list control groups and 57 participants completed the study. The study’s primary aim was to observe change in empathy. Participants completed pre- and post- intervention questionnaires as well as short questionnaires completed by participants and therapists after each session. The sessions were conducted at three training and therapy centers in Southwestern Germany. Some participants were familiar with each other from other programming at the site. They did not start other new therapies during the time of the study (Mastrominico et al., 2018).

#### Intervention of the Parent Study

The hour-long DMT groups of the parent study focused on mirroring activities and had 5–10 participants, a dance/movement therapist, and 1–3 research assistants. The groups followed the same structure every session including: (a) warm-up, (b) mirroring with a partner, (c) group mirroring of one participant’s movement, and (d) a closing with movement, verbal processing and a self-report questionnaire (for more specific intervention description see Koch et al., 2015; Mastrominico et al., 2018). For the partnered mirroring activity, the participants were instructed to pick a partner and choose who would be the first person to lead. This person was instructed to dance to one song, leading movements for their partner to follow. The second person was instructed to follow the movements keeping the feeling quality of the leader’s movements. Then the partners switched roles so that the second person took a turn leading the dance for the next song. The partners stayed together for one song of open-ended dancing during which they were instructed to “move as you want, but make sure you keep in contact with your partner.” The participants danced with different partners each week including other participants on the autism spectrum and research assistants, providing them with the opportunity to practice flexibly adapting their movement interactions to different partners.

### This Secondary Analysis

This secondary analysis received additional ethics approval from Drexel University IRB with a waiver of consent for the analysis of video data recorded as part of the parent study.

#### Participants in the Secondary Analysis

Of the participants in the parent study, the participants selected for this secondary analysis were those who were most often present and clearly visible with their partners in the videos of the partnered mirroring activity. The first author (EM) reviewed all the video from the parent study and noted the participants involved for each session in which a camera was focused on one particular dyad for the mirroring and both partners remained in the frame for at least 30 s. The participants selected for this secondary analysis were 14–42 years old including three white males and two white females. They had each attended between 5 and 10 sessions with video available from between 4 to 9 of these sessions (see Table 1).

**TABLE 1:** Participant demographics and sampling of video clips.

Participant	# of sessions attended	# of sessions recorded	# of video clips	Interactive video from which activity^a^	Diagnosis^e^	Age	Gender
Hans	10	8	31^b^	5 following, 1 open dance, 2 transition	Autistic disorder	42	Male

Lukas	9	9	34^b^	1 leading, 2 following, 6 open dance	Asperger syndrome, ADHD	14	Male

Karl	8	8	32	2 leading, 2 following, 2 open dance, 2 transition	Autistic disorder	21	Male

Julia	9	4	16	1 leading, 3 following	Asperger syndrome	26	Female

Anna	5	4	19^c^	5 leading, 1 following, 1 open dance	Autistic disorder	18	Female

Total			120^d^				

*All names are pseudonyms.*

*^a^Video clips selected for the most interactive behaviors could be selected from any of the following activities each session: following, leading, open-ended dance, or transitions between these three mirroring dances. This lists which activity the interactive video clips were selected from for each participant.*

*^b^Missing video clips due to videographer error or partners moving in-and-out of frame throughout the activity.*

*^c^Additional purposively sampled video clips of interactions added to increase sample size.*

*^d^Total number of videos do not add up to 132 as participants were occasionally partnered with each other.*

*^e^Self-reported diagnosis using ICD 10 terms for diagnosis closest to self-report.*

#### Selection of Video for the Secondary Analysis

The first author (EM) selected four 30-s video segments per participant per session. Three video clips were systematically sampled from the middle of the three partnered mirroring activities: leading, following, and open-ended dance. As these were of different lengths based on the music used that day, the time at the exact middle of each piece of music was calculated and the 15 s before and after that time were included in the 30 s clip. If one of the partners left the frame of the video for more than 2 s or a third person approached them, the video selection was shifted by as few seconds as possible to include the 30 s before or after this event.

A fourth, purposively sampled clip, was included to explore movement during interactions. For this, EM reviewed the video to notate the time of any occurrences of seven predefined interactive behaviors and selected the 30-s with the most of these interactive behaviors without overlapping with an already selected video clip. Interactive behaviors were defined as any of a list of seven observable behaviors that showed both partners responding to the other, such as turn taking, or behaviors, such as eye-contact, that are commonly understood to support social interactions. For the two participants who were only recorded for four sessions, EM reviewed the video for additional examples of their interaction, resulting in three additional interaction clips for one participant (Anna).

In 12 video clips, two of the participants from this secondary analysis were partnered with each other. Video samples were scored for each participant, making a total of 132 assessments of 120 video clips with between 16 and 34 video clips scored and described for each of the five participants (see Table 1).

### Measures

#### Synchrony

Synchrony was scored using Likert scales describing the percent of the time the partners were in a particular type of synchrony with each other (from 0 = *does not occur* to 4 = *76–100% of the time*). This initially included several forms of synchrony to differentiate between moving in time with each other (rhythmic synchrony) and moving at a slight delay (following); using the exact same shape (exact spatial synchrony) or similar but not exactly the same shape (approximate spatial synchrony); using the same feeling tone quality by matching characteristics of how they did the movement, such as matching the strength or lightness within the movement (effort synchrony); or moving in opposite directions (counter spatial synchrony). All synchrony scales were created for this study and based on the Fraenkel-Franks Index of Shared Behaviors (Fraenkel, 1983), the taxonomy of mirroring of Eberhard-Kaechele (2012), and theoretical assumptions around potential differences in individuals on the autism spectrum. Five forms of synchrony were dropped from the analysis due to low inter-rater reliability (following, approximate spatial synchrony, effort synchrony, and counter spatial synchrony) or high correlation with rhythmic synchrony (exact spatial synchrony). Only rhythmic synchrony was used for the final analyses and was defined as the “simultaneous movement of like or unlike body parts which begin and end simultaneously, and … move at the same rate” (Fraenkel, 1983, p. 38). For this study, this included single actions when both partners simultaneously changed the direction of movement, as well as longer sequences of movement.

#### Interaction Quality

Interaction quality was defined as the individual’s affective engagement with the partner together with the flowing quality of their interaction. This was measured using Garcia-Perez et al.’s (2007) scales of affective engagement and flow of the interaction. These 5-point scales include a rating guide which describes interactions at the different levels. Affective engagement describes engagement ranging from *no emotional connection* to *strong emotional connection* (Garcia-Perez et al., 2007, pp. 1314–1315). Flow of the interaction ranges from a *minimal degree of mutual exchange* to *flowing at a relaxed and steady pace* and was not scored for the videos of structured leading and following due to the lack of back-and-forth exchanges in these tasks. These scales were designed to assess the interaction in videos of interviews of adolescents on the autism spectrum or intellectual disability, with Kappa values showing moderate inter-rater agreement in Garcia-Perez et al.’s (2007) study.

#### Supplemental Scales

Additional scales were used to provide context and check for participation. Raters selected from statements describing the degree to which participants were engaged in moving, leading, or following. This showed that participants led and followed mirroring as assigned and were moving throughout most of the open-ended dance. This was not used for further analysis.

Participants’ distraction and use of restrictive/sensory seeking movements was scored on 3-point scales. To determine the level of distraction, the raters were instructed to observe when participants turned their heads to look away, interacted with other people/objects, and other non-verbal cues of shifting attention away from the partner. Restrictive/sensory seeking movements was scored only when the movements appeared to be sensory seeking or otherwise restrictive, and not for repetition in the dance since inexperienced dancers can quickly run out of movement ideas. These were examined for each participant over time and compared to related qualitative themes in each video for each participant.

### Rating Procedures

Two sets of raters were employed to score the different measures and write descriptions of the movement and interactions in the videos (see Table 2 for an overview of video rating). All raters were trained on the scales using videos of other participants of the parent study. The order of the videos for each participant were randomized using an online randomizer so that the raters were blinded to session number. All raters were given the videos to watch and then score in word documents which started with the scales they were assigned and then spaces to freely describe the movement and the interaction in the video clip. During training, raters practiced writing brief narrative descriptions of a few sentences each for the movements and interactions they observed in the video.

**TABLE 2:** Mirroring activities, video sampling, and rating.

Video type	Activity	Video sampling method	Quantitative and qualitative data collected	Raters
Leading	Leading the partner when instructed to mirror each other	Systematic sampling: 30 s taken from the middle of the activity	All scales rated Short descriptions of participant’s movement and interaction written by all raters	Three interaction quality raters for all video clips One primary synchrony rater for all segments, a second synchrony rater on 20% of the video clips
Following	Following the partner when instructed to mirror each other			
Open-ended dance	Open-ended dance with instructions to “stay in contact with your partner,” not assigned leading or following role		All scales rated Short descriptions of participant’s movement and interaction written by all raters Additional page with movement characteristics of interest completed by synchrony scale raters	Three interaction quality raters for all video clips Two synchrony raters for all video clips
Interactive behaviors	Any of the above mirroring tasks	Purposive sampling: 30 s selected for most interactive behaviors		

#### Interaction Quality Raters

Three raters scored all the videos for affective engagement, flow of the interaction, distraction, and restrictive/sensory seeking movements. The third rater was added when it became clear that one of the first two raters had rated the distraction and restrictive/sensory seeking movements scales incorrectly and was no longer available to correct this error. Quantitative data analysis of interaction quality used the average score of the three raters.

#### Synchrony Raters

Three different raters, master’s level DMT students, rated the video segments for synchrony and described characteristics of the movement in addition to the general qualitative descriptions. One rater was assigned as the primary rater for each participant and scored all of the videos for that participant, while a second rater scored a randomly selected 20 + % of the leading and following videos and all the open-ended dance and interactive videos of that participant. The synchrony scores were averaged for the quantitative analysis in sessions with two raters and the primary rater’s scores were used in sessions with only a single rater scoring synchrony. These raters were instructed to use the standardized movement language of Laban Movement Analysis (LMA) or the Kestenberg Movement Profile (KMP) in their narrative descriptions of the movement in the video. For the open-ended dance and interaction clips, they were provided with an additional chart to note the obvious use, or absence of, specific categories of movement qualities to elicit more information on potentially relevant characteristics of the participants’ movement during interactions.

### Data Analysis

The scores on the quantitative measures were examined for change over time and correlations between the variables. The descriptions of the participant’s movement and interactions were examined using a qualitative descriptive analysis. The quantitative and qualitative strands were compared for a richer analysis of the results.

#### Quantitative Analyses

For the first stage of analysis, graphs were created for each participant with the scores on each scale shown over time. This included separate graphs for each video segment type. These graphs were inspected for any observable trends over time, patterns that repeated across multiple individuals, and potential relationships between the variables. For those participants for whom visual inspection of the graphs suggested a possible impact of time on any scale, their scores were correlated with session number using Spearman’s rho to test for change over time. To test for a relationship between synchrony and interaction quality, the synchrony, affective engagement, and flow of the interaction scores were correlated using Spearman’s rho. These correlations were run for each individual participant. To test for potential patterns in the correlations across this small sample of five participants, paired *t*-tests were run using the different correlation values for each participant.

#### Qualitative Descriptive Analysis

A qualitative descriptive analysis was conducted to provide a rich description of the movement and interactions with only a low level of interpretation of the data (raters’ descriptions of the movement and interactions). For this analysis, the first author (EM) used MAXQDA data analysis software to undertake thematic and content analysis procedures to search for commonalities, differences, and relationships between themes within and across participants. Participants were listed as separate cases in MAXQDA with all rater materials organized by session to allow for analysis over time. Braun and Clarke (2006, p. 87) described this semantic level thematic analysis in 6 phases of: (1) familiarizing yourself with the data (2) generating initial codes, (3) searching for themes, (4) reviewing themes, (5) defining and naming themes, and (6) producing the report.

To conduct this analysis EM first read all the descriptions for each participant (phase 1). Then (phase 2) EM coded the descriptions using both (a) *a priori* codes based on theoretically relevant concepts and (b) codes that emerged through engagement with the data. *A priori* codes were created for: the constructs measured by the quantitative scales (forms of synchrony and delayed following, affective engagement, flow of the interaction, repetitive or sensory seeking movements, attention and distraction), motor skill and coordination in the mirroring task, and LMA and KMP movement quality terms. After it was determined that the raters mostly listed LMA and KMP movement qualities without any further description of the context or interaction at that moment, these were assessed for frequency of each term and then collapsed into larger categories referring to descriptions of any movement qualities rather than coding individual movement qualities. These new codes included: movement qualities described as present, absent, matching or mismatching the partner’s movement qualities, and movement described in relation to the interaction. For further codes, EM read the descriptive data to identify other topics that recurred across segments, topics on which there was agreement or disagreement between raters on a segment, or subcodes that gave further nuance to the *a priori* codes. This resulted in both novel codes and subcodes. In order to remain close to the research question, the coding and search for themes from these codes (phase 3) was focused on identifying potential themes or patterns in the descriptions of the interactions, the relationship between the movement and interaction, changes in movement qualities or engagement, and non-verbal cues of engagement or lack of engagement in the interaction. Coding and thematic analysis was conducted first by participant, with an exploration of themes within the individual’s data. As new codes and themes were considered, EM returned to previously analyzed participants’ data for review using the novel codes and themes (phase 4). After this individual analysis, codes and themes were examined for patterns or discrepancies across the five participants and refined into a final set of themes (phase 5).

A peer reviewer with experience working as a dance/movement therapist with this population examined the coding scheme and performed a data audit by reviewing the coding of the qualitative descriptions of three videos. The peer reviewer and EM discussed the coding scheme until they reached agreement that the coding scheme: (1) captured the movement and interaction information included in the descriptions, and (2) reflected dance/movement therapy work with this population.

#### Mixed Methods Synthesis

The qualitative and quantitative strands of this study were integrated throughout the design, analysis, and final integration of the results with findings from each strand influencing decisions on further analyses in the other strand. Qualitative codes were displayed in charts showing their occurrence over time to visually compare these with graphs of the participant’s quantitative scores. Similarities were used to help explain the relationships between the movement and the interaction, and inconsistencies guided a return to each form of data in search of possible explanations for the differences. For instance, the qualitative findings describing different approaches to the different mirroring tasks supported the decision to run correlations separated by mirroring task and the combinations of the more structured leading and following mirroring in contrast to the less structured open-ended dance and interactive videos.

## Results

The participants were observed to approach the three mirroring tasks differently and each participant had their own unique movement patterns (see example descriptive quotes in Table 3).

**TABLE 3:** Descriptions of how each participant tended to approach movement and mirroring.

	General approach to movement or mirroring	Example quotes
Hans	Often watched others in room for ideas when leading and during the open-ended dance, frequently led slow arm movements.	When following: “A big smile blooms on his face during the quicker movements. As the movements slow down, his attention stays on [his partner], but more toward her feet and his face becomes less emotionally expressive.”
Lukas	Led large energetic movements while looking slightly off to side, mostly leaping side-to-side with other movements periodically thrown in.	When following: “[Lukas] follows the movements of [his partner] exactly. The hand form is also exactly copied.” During open-ended dance: “[Lukas] seems to be in his own world, enjoying the dancing but unaware of those around him, including his partner.”
Karl	Often led uncoordinated movements that appeared hard for partners to follow, such as moving arms while doing a forward-backward motion of his pelvis that made him appear unstable. Followed example of others in his group to try to poke, tickle, or kick at others to briefly engage.	When leading: “[Karl] leads [his partner] through movements that are typical of him: arms up and down, arms from side to side while bouncing his weight on the balls of his feet. [Karl] and [his partner] then exchange a karate-like move.”
Julia	Used a large range of varied movements when leading and dancing on her own, frequently inattentive to her partner during the open-ended dance.	During open-ended dance: “creates a lot of different movements, turning, bending, moving upper body from side to side, seems to enjoy her creativity and freedom to move.”
Anna	Often led a series of gestures depicting sports and every day actions with interruptions to make repetitive movements of one hand against the other. Her partners generally waited until she stopped and returned to lead other actions.	When leading: “shows a whole repertoire of movements with meaning, most of them everyday movements (washing hands, eating, drinking, brushing teeth, etc.). No abstract movement in this section.”

### Inter-Rater Reliability (Observer Agreement)

Three raters scored all the videos for interaction quality with strong inter-rater reliability on affective engagement ICC(2, 3) = 0.833, 95% CI [0.777, 0.877] and flow of the interaction ICC(2, 3) = 0.808, 95% CI [0.711, 0.876]. There was moderate to strong agreement between the two raters of the secondary scales on distraction ICC(2, 2) = 0.713, 95% CI [0.595, 0.796] and restrictive/sensory seeking movements ICC(2, 2) = 0.663, 95% CI [0.525, 0.761]. Because different pairs of raters scored each participant’s videos for synchrony, inter-rater reliability was calculated per participant with all rater pairs showing strong agreement on scores of rhythmic synchrony (see Table 4).

**TABLE 4:** Inter-rater reliability on the rhythmic synchrony scale by participant.

participant	Hans	Lukas	Anna	Karl	Julia
Inter-rater reliability, synchrony	ICC = 0.862** *n* = 19 CI[0.642, 0.947]	ICC = 0.742** *n* = 23 CI[0.392, 0.891]	ICC = 0.793** *n* = 13 CI[0.321, 0.937]	ICC = 0.726** *n* = 21 CI[0.324, 0.889]	ICC = 0.928** *n* = 12 CI[0.749, 0.979]

ICC all (2,2). There were 2 raters for the synchrony scales per participant. One primary rater scored all of the videos for that participant and a second rater scored at least twenty percent of the videos. For this table n = number of videos scored by two raters. The primary and secondary raters varied by participant. **p < 0.01.

### Attention/Distraction and Restrictive/Sensory Seeking Movements

Only two participants (Hans and Karl) were scored as distracted for more than a few seconds, for a total of 13 clips. Only one participant (Anna) was scored as engaged in clear restrictive/sensory seeking movements that distracted from the partnered movement.

### Change Over Time

**FIGURE 1:**
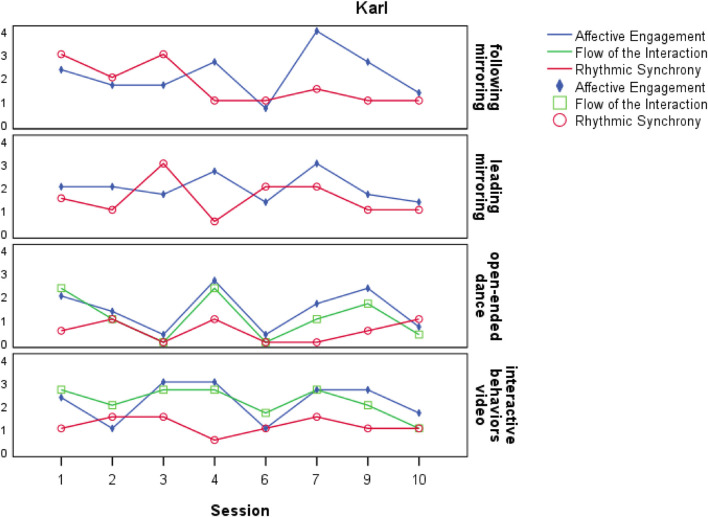
Change in synchrony and interaction quality over time for Karl. Change over time for Karl showing affective engagement, flow of the interaction, and synchrony with his partners each week. Graphs separated by video type with leading, following, open-ended dance, and a video selected for the most interactive behaviors displayed during one of these tasks. Flow of the interaction was not scored in the following and leading videos.

Four of the five participants were paired with different partners in almost every session and their scores either remained relatively stable or varied week by week, showing no observable trends in any variable over time (see Figure 1 for an example and Supplementary Materials for graphs of change over time for each participant). The fifth participant, Hans, was partnered with the same dance/movement therapy student for five of the eight recorded sessions. His affective engagement increased over the five open-ended dances with this partner *r*_*s*_(3) = 0.900, *p* = 0.037 when correlated by session number (see Manders et al., 2021, for an in-depth analysis of this case). This increase was not observed when the sessions with other partners were included in the analysis.

### Correlational Analyses

Correlations between synchrony and interaction quality were calculated per participant (see Table 5). With only 4–9 videos per video type for each participant, these correlations were run with the video types combined into (1) the more structured leading and following segments and (2) the less structured open-ended dance and interactive segments. Due to the non-independence of the 12 videos in which two of these participants were partnered with each other, the videos were assigned to just one of the two participants using stratified randomization. This meant that each participant was randomly assigned one of the videos of each type. With the small sample sizes, few of these correlations were significant. The correlation coefficients were, however, larger for all participants in the open-ended dance + interactive segments than the leading + following segments. A paired *t*-test of the correlation coefficients for each of the five participants showed a significant difference between the average correlation coefficients for the leading + following segments (*M* = 0.016, SE = 0.187) and the open + purposive segments (*M* = 0.479, SE = 0.1092), *t*(4) = 2.840, *p* = 0.047, 95%CI[0.010, 0.917], indicating that the correlation between Synchrony and Affective Engagement was significantly stronger in the segments with less structure or more interaction. Another paired *t*-test of the correlation coefficients for the open-ended dance and interactive video clips for each participant showed a significantly stronger correlation between synchrony and flow of the interaction (*M* = 0.611, SE = 0.105) than synchrony and affective engagement (*M* = 0.479, SE = 0.109), *t*(4) = 4.427, *p* = 0.011, 95%CI[0.049, 0.215].

**TABLE 5:** Correlations between synchrony and interaction quality for each participant.

Participant and video type	Synchrony and Affective Engagement			Synchrony and Flow of the Interaction		
	Spearman’s Rho	n	Sig.	Spearman’s Rho	n	Sig.
Hans leading + following	0.019	15	0.947			
Hans open-ended dance + interactive^a^	0.496	14	0.072	0.634*	14	0.015
Karl leading + following	0.104	14	0.724			
Karl open-ended dance + interactive^a^	0.246	14	0.397	0.425	14	0.130
Lukas leading + following	0.122	17	0.642			
Lukas open-ended dance + interactive^a^	0.357	17	0.160	0.393	17	0.119
Julia leading + following	0.493	6	0.321			
Julia open-ended dance + interactive^a^	0.883*	6	0.020	0.985**	6	<0.001
Anna leading + following	−0.660	7	0.107			
Anna open-ended dance + interactive^a^	0.413	10	0.236	0.619	10	0.057

*Correlations were calculated using spearman’s rho. The structured leading and following segments, and the less structured and interactive segments, were combined to increase the n for each correlation. Flow of the interaction was not scored in the following and leading video segments. For the 12 videos with two participants paired with each other, one video of each type was randomly assigned to each of the two participants to avoid using the scores from the same video twice.*

*^a^Video clips selected for the most interactive behaviors in the 30-s segment of the leading, following, or open-ended dance based on a predefined list of interactive behaviors.*

*p < 0.05, **p < 0.01.

### Qualitative Themes

Raters described participants as having different patterns of interaction with different partners, and suggested that participants may have had personal preferences for some partners. The five participants were each described as engaging and moving in their own ways, and there were also patterns in the descriptions that recurred across multiple clips and participants. Quotes from the raters’ descriptions were corrected for spelling and participant pseudonyms were added for ease of reading.

#### Movement Qualities

The participants varied in their personal movement style, motor skill, ability to match their partner’s movements, and ability to coordinate their upper and lower bodies. Some participants used a large variety of movements and little hesitation, while others tended to do repetitive steps, watch others for ideas, or pause between movements. This showed a wide range in their ability to create a varied or continuous flow of movement for a dance. As a group, the participants did not lack any of the LMA or KMP movement qualities, although some movement qualities were noted less frequently (see Manders et al., 2021, for further analysis of movement qualities). Challenges with coordination, integration of movement through the body, and balance occurred across several of the participants.

#### Attention to Task vs. Social Attention and Engagement

Raters frequently described participants as following the instructions to lead, follow, or dance, but without much social connection to their partner. They were observed to look down or slightly off to the side of their partners while mirroring, and when they appeared to look toward their partner’s face, they showed few facial expressions, instead seeming to mostly look to check in when there were changes in the movement. For example, “[Anna] does not make eye contact with [her partner] throughout the time, but looks toward her feet and glances upward occasionally to follow the motions.” In another example, a rater observed that during the open-ended dances, Julia could be entirely engaged in her own dance as she: “dances very enthusiastically, bending her knees, tilting from side to side, turning, and moving her arms in many different ways. Although both appear to be enjoying themselves and dance the entire segment, there is no visible interaction between [them].”

At times, some participants did appear to lose their attention to the task and move “distractedly” by shuffling their feet, stepping from side-to-side, or briefly stopping moving. Anna was the only participant who stopped mirroring to perform obvious sensory seeking or repetitive behaviors, and she was frequently described as appearing distracted or uncertain about the task: “it appears that [Anna] does not quite know what movements to do, and. itches herself on the hand or is fidgety and easily distracted.”

#### Limited Development of Social Engagement

Smiling was one of the more commonly reported interactive behaviors in the videos. Four of the five of the participants were described (by one or more raters) as smiling, laughing, or sharing smiles with their partners, in at least half of the video clips. The participants sometimes smiled immediately after looking up and giving eye-contact, or after their partner smiled. Some participants appeared to smile more with preferred partners or in response to specific events in the activity such as unexpected changes, challenging or playful movements, and accidental missteps. For example, in one clip Karl followed Julia through a variety of movements including following her in turning to face away from each other so they could not see each other. The rater noted that “when [Julia] turns back around to face [Karl], [Karl] does not realize it for a second, and when he does he hops around, smiling at his mistake. [Julia] smiles back at him, and they continue through the motions.”

While the participants all laughed or engaged in brief interactions with their partners at times, these tended to be brief and infrequent. As one rater put it (in Hans’s first session): “they are somehow connected and interact, but do not develop something together.” Karl also showed this pattern when he “playfully ‘kicks’ [his partner], to which [his partner] smiles and acts as though he will fall over. [Karl] smiles, but the interaction then fades.” When his usual partner led variations on a playfully challenging movement theme, Hans laughed and seemed “to be having a good time,” but he did not appear to try to develop these interactions himself.

#### Attuning to the Partner

While the participants were most often described as attending to the task rather than the person of their partner, the raters also several times noted that participants sometimes appeared to adjust their movements according to their partner’s needs. This included seeming to wait or slow down for a partner who was having trouble following, simplifying movements, verbally explaining a movement, or testing a partner’s ability to follow. Multiple raters noted that Lukas seemed to sometimes go slower through variations on his typically quick movement sequence with partners who had difficulty keeping up. Other participants at times appeared to wait for each other: “When it takes [Karl] a second to get a motion, [Julia] is aware of that and waits for [Karl] to get it before moving on.” These behaviors were not necessarily accompanied by other cues that the participant was adapting to their partner or trying to engage, and it generally did not develop into a reciprocal interaction.

### Mixed Methods Synthesis

Quotes from the qualitative themes were arranged by video segment to allow for comparison to the graphs of the scores on the quantitative scales. The overall qualitative themes and descriptions of each participant’s common patterns were also reviewed next to the participant’s quantitative results.

While the scores on the distraction scale and descriptions of participant’s attention both indicated that the participants were more often attentive than completely distracted, descriptions of distraction did not always correspond with higher distraction scores in that video. There were more descriptions of aspects of lack of attention to the other person than there were videos scored for distraction due to the participant turning away, or shifting their attention to something or someone else. The descriptions added nuance to the topic by pointing out distinct aspects of attention and distraction, making it clear that attention to the task was not necessarily associated with attention to the other person. The participants did not always look at their partner if it was not essential to the task. They did generally keep sufficient attention near their partner to follow large body movements or lead movements themselves.

The descriptions of the participants’ different patterns of engagement with different partners, and the partners’ own varying degrees of responsivity, support the interpretation that the weekly variation in quantitative scores may have reflected the different partnerships. The impact of the intervention could therefore not be isolated, except in the case of the one participant who had several sessions with the same partner.

Descriptions of participants’ different patterns of engagement in the leading, following, or open-ended dance tasks supported the decision to run the correlational analyses in the combinations of leading and following in contrast to the open-ended dance or interactive videos. These descriptions help explain the pattern of lower correlation coefficients between synchrony and interaction quality in the leading and following videos when participants focused on the task itself and not engagement with the other person.

## Discussion

The most striking findings of this pilot study of five participants on the autism spectrum were the extent of the difference between engagement in the instructions of the task and engagement with the partner, and the suggested complexity of the relationship between synchrony and interaction quality. Distinctions between engagement in the task and engagement with the partners were observed in the qualitative and mixed methods analyses of the patterns of attention and distraction, and the qualitative descriptions of the movement and interactions. In addition, while some participants varied their movement phrases, all of their interactions were brief. The participant’s partners had their own impact on the interactions. Change over time was only noted in Hans, the one participant who had the same partner for 5 weeks, and only for the open-ended dances when this was calculated for just the sessions with this partner. In an unexpected finding, synchrony and affective engagement were more strongly positively correlated in the less directive open-ended dance and videos selected for more interaction than in the more structured leading and following mirroring tasks. While a larger study would need to confirm this finding, for these participants, this pattern was seen in larger correlation coefficients in the less directive or interactive videos for all five participants with a paired *t*-test showing this to be a significant difference. This suggests that the role that synchrony plays in interactions may vary depending on context. Increasing synchrony through proscribed leading and following mirroring tasks on their own may not be sufficient to address the non-verbal social challenges in individuals on the autism spectrum. The stronger correlation across the five participants between synchrony and interaction quality in the open-ended dance and interactive segments suggests that it may be useful for further studies to explore the use of interactional synchrony in complex social situations.

Within this group of five very different participants, the relationship between synchrony and interaction quality appeared to be more complex than expected: varying between type of mirroring activity. This structured mirroring task was designed to encourage interpersonal synchrony, so when completed without additional interactive behaviors or signals of affective engagement, it is not surprising that synchrony would be observed without necessarily having a strong relationship to interaction quality. Although the sample sizes of the videos per participant were small with few correlations reaching significance, the larger positive correlation coefficients between synchrony and affective engagement or flow of the interaction in the open-ended dance or interactive videos may reflect an impact of synchrony on social engagement in these contexts. Marsh et al. (2013) claim that “the ability to time, coordinate, and *flexibly adapt* [emphasis added] our movements with others, may underlie or contribute significantly to our ability to engage others socially” (p. 7). Such flexible adaptations were not necessarily required for leading and following mirroring with its dictated roles, while being essential for the back-and-forth of interactions. This would be in line with the suggestion that typical patterns of interpersonal synchrony depend on the context, and individuals on the autism spectrum may find it challenging to adapt in real time during complex interactions (Delaherche et al., 2013; Marsh et al., 2013; Fitzpatrick et al., 2018). It may also be that structured mirroring with assigned roles and open-ended dance address different aspects of social interaction. Leading and following may reflect intentional synchrony, which Fitzpatrick et al. (2018) suggest may relate to social action and attention, while synchrony during interactions or the open-ended dance may have reflected spontaneous synchrony, which Fitzpatrick et al. (2018) propose may be related to implicit social knowledge and understanding of intentions. This would suggest that the different stages of mirroring and the open-ended dance may address different aspects of social competency. On the other hand, since the segments with the most interactive behaviors were selected from all of the mirroring activities, it may be that any of these could be developed into this more complex stage of interaction. For some participants, interactive behaviors were found fairly evenly across the mirroring tasks with videos selected from each task. Other participants showed most of their interactive behaviors in just one of the three mirroring activities. What is the most likely to develop into interaction may depend on the individuals and their partners.

Given that the open-ended dance is a more complex scenario for social engagement, and Hans showed increased affective engagement in the open-ended dance over time with the partner who repeatedly led him in a playful movement challenge, it is interesting to explore this more closely. It may be that Hans’ personality and motivation to follow made him more receptive to this playful movement challenge in mirroring, or the familiarity with the partner or the task of mirroring supported his engagement. Further research should investigate if interventions that use more spontaneous, playful, and flexible structures can support participants in adaptively using synchrony in more complex social situations. Such interventions may need to titrate or build up to these less structured tasks as individuals on the autism spectrum frequently seek routines and can become anxious without clear or familiar structures. Hans’ increased affective engagement in the open-ended dance was also likely supported by his initial practice with more structured leading and following as he was described as most affectively engaged when following his usual partner in her playful movement challenge, and he appeared to enlarge his movement vocabulary over the 10 sessions by repeating others’ movements.

The theoretical assumption that the use of a “body close” task that required attending to, and coordinating with, another person’s movements would aid socialization through an embodied experience of sharing in the emotional tone and qualities of the movements with a partner, did not appear to be supported in this study. Unlike the participants in this study, people with strong prosocial skills generally engage in mirroring as a social activity with non-verbal cues of togetherness, play, shared affect, positive affect, and referencing each other even in the more structured roles of leading and following (Feniger-Schaal et al., 2018). While prior studies have found that children on the autism spectrum increase their social interactive behaviors after being mirrored (Field et al., 2013; Samaritter and Payne, 2017), these studies were of children and measured the frequency of specific behaviors rather than a more subjective overall assessment of affective engagement, potentially accounting for the difference in the results. The participants in this study may have been too old for this embodied sharing of states to work, or for it to work in such a short period of time, as infants with a typical level of preferential attention to faces and a strong pull toward intersubjectivity would have years of practice in this type of coordinated awareness to develop social skills. The participants’ limited eye-contact and low levels of attention to their partners’ faces may also have contributed to their limited emotional matching to build social understanding and successful engagement. When they looked at their partners, the participants appeared to use more “checking” or “orienting” type looks toward their partner than “sharing” looks, whereas more “sharing” type looks may have made the mirroring appear more affectively engaged and responsive (Hobson and Hobson, 2007). This may have been related to the social complexity of the mirroring dance as eye gaze studies have found individuals on the autism spectrum may show differences in preferential observation of faces specifically in more complex situations (Guillon et al., 2014). Larkin et al. (2015) similarly found that in play with caregivers, children on the autism spectrum showed higher levels of shared attention to the task and “coordination of actions” compared to the control group’s higher levels of “coordination of intentions” and attention to the other person. This focus on the essential elements of the task rather than the other person may be an adaptive skill for those who find social interactions challenging. Little et al. (2015) hypothesized that for individuals with high attention to detail, a focus on the completion of the task may allow them to participate in more extracurricular activities, while avoiding some of the anxiety around the social aspects of the activity.

Despite their challenges, the descriptions of the movement and interactions suggested that some of the study participants at times attuned and adapted to their partner’s movement needs, abilities, or interests. This did not necessarily lead to longer interactions as they appeared to miss other critical aspects of initiating and maintain an interaction such as first capturing their partner’s attention, encouraging, or communicating with their partner. While this suggests a certain level of awareness of the partner and the partner’s perspective, these adaptations were infrequent and not always effective. An initial desire to repair the interaction might have been hindered by limited skill to flexibly try another method or scaffold a sufficient number of these adaptations or attempts at communication to be successful. The specificity of this challenge to complex social interaction was demonstrated in the participants who regularly attended to their partners, initiated social engagements, or creatively developed varied movement phrases: even with demonstrated skills in attention, social initiation, or movement development, they still had only brief or repetitive social interactions with their partners. The participants in Edwards (2015) DMT group described a similar challenge, stating that they could remain aware and responsive to each other’s sensory needs within the group while struggling in less supported everyday situations. Therapists may therefore want to support clients to strengthen these initial impulses to adapt to the partner and combine them with communication or other non-verbal cues within increasingly more complex social situations.

### Limitations

There were several limitations in this exploratory secondary analysis. First, there was a small sample size of only five participants, and even for these individuals, video was unavailable for some of their sessions. The limited video data from the parent study made it impossible to select a homogeneous group of participants for the secondary analysis. These preliminary results and themes should therefore be considered in relation to these particular individuals and cannot be generalized to others.

The use of secondary video data meant that this study had no control over the design or implementation of the mirroring intervention. Most participants had different partners on most weeks, including both research assistants and others on the autism spectrum. Thus, partners varied greatly in their social and movement abilities, and this may have obscured the effects of the intervention. Given the large differences in motor skill and movement repertoire between the participants, it is possible that the relationship between movement synchrony, interaction quality, and other features of the movement may vary dependent on motor skill, however, the small number of participants prevented any subgroup analyses in this study.

The qualitative findings may have been influenced by the fact that the raters scored the quantitative scales first, although this may be considered similar to an interview guide designed to elicit qualitative data relevant to the question. While all raters were fluent in English, the fact that English was a second language for most of the raters may have limited the richness of the descriptions. The qualitative strand was further limited by the format of the rating document as this provided separate areas for discussing movement, the interaction, and suggested categories for observation of the movement qualities by LMA or KMP terms. This led to lists of KMP and LMA movement terms more often than rich descriptions linking the specific movements into the context of the interaction. While the raters were blind to the session number when scoring the video, the first author (EM) was aware of the session order during analysis and this awareness may have influenced her data coding choices.

Despite the high interrater reliability, the use of scales (affective engagement; flow of the interaction) originally designed for use with verbal interviews in a DMT context may have compromised their validity. This issue of context was noticeable for the flow of the interaction scale: it was not rated for the videos of leading and following due to challenges in interpreting this scale when the participants were continuously connecting by moving together, but not making any attempts at back-and-forth interaction. The low inter-rater reliability of several of the synchrony scales leading to them being dropped from further analysis suggests that these may not have been sufficiently well defined or reflect insufficient rater training for these scales. For 12 videos two participants in this secondary analysis were partnered with each other meaning that while these were scored and described for each participant, they were not independent of each other. Looking at the same interaction twice may have impacted the results and qualitative findings. When these videos were each randomly assigned to only one participant for the correlational analysis, it reduced the problem of non-independence, but decreased the sample size of videos.

The study was limited in its reliance on observer’s assessments without obtaining the participant’s perspectives on their emotional engagement and their experience of connection to their partners. It has been reported that while individuals on the autism spectrum may at times appear disinterested in the other, this may be unintentional (Causton-Theoharis et al., 2009). While the participants’ perspectives were outside the scope of this study, the moments when some participants appeared to partially adapt to their partner’s abilities suggest that there were times when the participants may have experienced empathy or concern for their partner that they did not fully communicate. The use of mixed methods research with multiple perspectives and points of data on each participant including both qualitative and quantitative analysis of 120 video clips, was, however, a strength of this study.

### Research and Clinical Implications

While it may be tempting to try to *teach* individuals to develop the interaction by breaking it down into instructions, the different correlations between synchrony and affective engagement in the different segments, combined with Fitzpatrick et al.’s (2018) findings showing distinctions between spontaneous and intentional synchrony, suggest that this most likely needs to be practiced in more open-ended and spontaneous contexts. As participants on the autism spectrum may feel anxious without a predictable structure, it may be useful to introduce synchrony and model extending movement-based interactions within a more structured task, such as leading or following, however, this needs to be accompanied with contexts that allow for more flexibility to mimic typical interaction complexity for these skills to support successful social engagement. To do this, clinicians may need to start with modeling developing interactions and may want to emphasize playfulness and intentional surprises to engage clients and build their tolerance for flexibility in interactions.

As the participants of this study engaged more with certain partners than others, interventions should respect and make use of personal preferences, prior relationships, and the relative interactive and movement skill of the partners. Further research should explore the possible benefit of starting with partnering with a trained dance/movement therapist prior to pairing individuals with peers to give participants the experience of having the partner affectively engage while attuning to them before seeing if they can follow this model on their own with their peers. While the participants in the parent study were frequently partnered with others on the autism spectrum (Mastrominico et al., 2018), the participants in the pilot study were most often partnered with student research assistants (Koch et al., 2015) and this difference may have been a factor in the stronger findings of the pilot study. For this secondary analysis, the fact that the participant with five sessions with the same partner did show some improvement over time in the open-ended dance segments with this particular partner, suggests that further investigation into mirroring with consistent partners would be worthwhile. Further study may be particularly useful as this improvement was seen in the more flexible, and therefore complex, context of the open-ended dance.

Given the social challenges and the tendency to follow social skills as rules by individuals on the autism spectrum, it would be interesting to investigate if this apparently stronger correlation between synchrony and interaction quality in the structured leading and following clips compared to the interactive, and open-ended dance clips may be unique to those on the autism spectrum. Therefore, further research is needed to explore this distinction with a larger sample with individuals on the spectrum as well as others not on the autism spectrum. Larger studies could furthermore examine what may be driving this correlation in the open-ended dance, and if this pattern of varying correlations in structured and more flexible contexts holds for other types of tasks that require interpersonal coordination between partners. In addition, the stronger correlation between synchrony and flow of the interaction than between synchrony and affective engagement suggests that flow of the interaction may be more responsive to interventions addressing interpersonal synchrony. There is a need for further research to better understand the role of the flow of the interaction on the overall social success of individuals on the autism spectrum. Such research would benefit from an operational definition for the flow of the interaction in scenarios and movement-based activities that may involve coordinated action but not a back-and-forth exchange. It would be useful for larger studies to continue to explore possible differences in the characteristics of the movement with subgroups of individuals with and without challenges in coordination, fine, and gross motor skills.

## Conclusion

There is a need for more research on the creative arts therapies and movement-based interventions for interactional synchrony and social competency in people on the autism spectrum. This secondary mixed methods video analysis added an operational definition of rhythmic synchrony and description of the participants’ strong task focus to the exclusion of the social aspects of moving together in more structured activities. This study followed five participants over 4–9 sessions of mirroring in dance/movement therapy, showing some preliminary themes and results in this small group of heterogeneous participants on the autism spectrum. These results included stronger correlations between synchrony and affective engagement in the less structured open-ended dance and interactive clips than the structured leading and following for each participant. For these individuals, it appeared that rhythmic synchrony did not support rapport when they were focused on a structured task that did not require social engagement. This should be investigated further with a larger sample size. Additionally, given that synchrony was more strongly correlated with flow of the interaction than affective engagement, it would be useful to explore how rhythmic synchrony supports a flowing interaction when the two individuals are not engaged in exact mirroring of actions, and rather in an interactional exchange. Consequently, there is a need to study movement interventions for interactional synchrony that prioritize spontaneity, open-endedness and creativity. It would also be important to explore whether it is possible to support participants’ nascent awareness of the other and apparent adjustments for the other’s abilities at a bodily level to help individuals on the autism spectrum develop this into longer, multidimensional, social interactions. The authors recommend that mirroring and synchrony-based interventions maintain the creativity and joy of dance when helping individuals on the autism spectrum explore the complexities of the dance of social interaction.

## Data Availability Statement

The original contributions presented in the study are included in the article; further inquiries can be directed to the corresponding author/s.

## Ethics Statement

The studies involving human participants were reviewed and approved by the Drexel University IRB (for current study) and Medical Faculty of the University of Heidelberg Ethikkommission (approval of parent study with informed consent given for parent study). Written informed consent from the participants’ legal guardian/next of kin was not required to participate in this secondary analysis in accordance with the national legislation and the institutional requirements.

## Author Contributions

EM conducted the secondary analysis as part of her dissertation and wrote the first draft of the manuscript. SG served as primary advisor on the dissertation. SK served on the dissertation committee and made significant contributions to the writing of this manuscript. EG served on the dissertation committee and gave input into the manuscript. MP served on the dissertation committee and supported the statistical analyses. KF served on the dissertation committee and supported the qualitative analyses. TF acquired funding for the parent study. TF and SK designed and conducted the parent study. All authors reviewed and gave feedback on the final version.

## Conflict of Interest

The authors declare that the research was conducted in the absence of any commercial or financial relationships that could be construed as a potential conflict of interest.

## Publisher’s Note

All claims expressed in this article are solely those of the authors and do not necessarily represent those of their affiliated organizations, or those of the publisher, the editors and the reviewers. Any product that may be evaluated in this article, or claim that may be made by its manufacturer, is not guaranteed or endorsed by the publisher.

## Footnotes

1 The term “individual on the autism spectrum” will be used throughout this paper as a form of person-first language that is generally considered less offensive by those to whom it refers than “person with autism” (Botha et al., 2021).

## Abbreviations

ASD: Autism Spectrum Disorder
DMT: Dance/Movement Therapy
KMP: Kestenberg Movement Profile
LMA: Laban Movement Analysis.
