# Supplementary material for: The Mirroring Dance: Synchrony and Interaction Quality of Five Adolescents and Adults on the Autism Spectrum in Dance/Movement Therapy
Source: Front Psychol. 2021 Oct 14;12:717389. doi: 10.3389/fpsyg.2021.717389 (PMC8551749; doi:10.3389/fpsyg.2021.717389)
Supplement: Supplementary file 1 [file Data_Sheet_1.pdf]

*Supplementary Material*

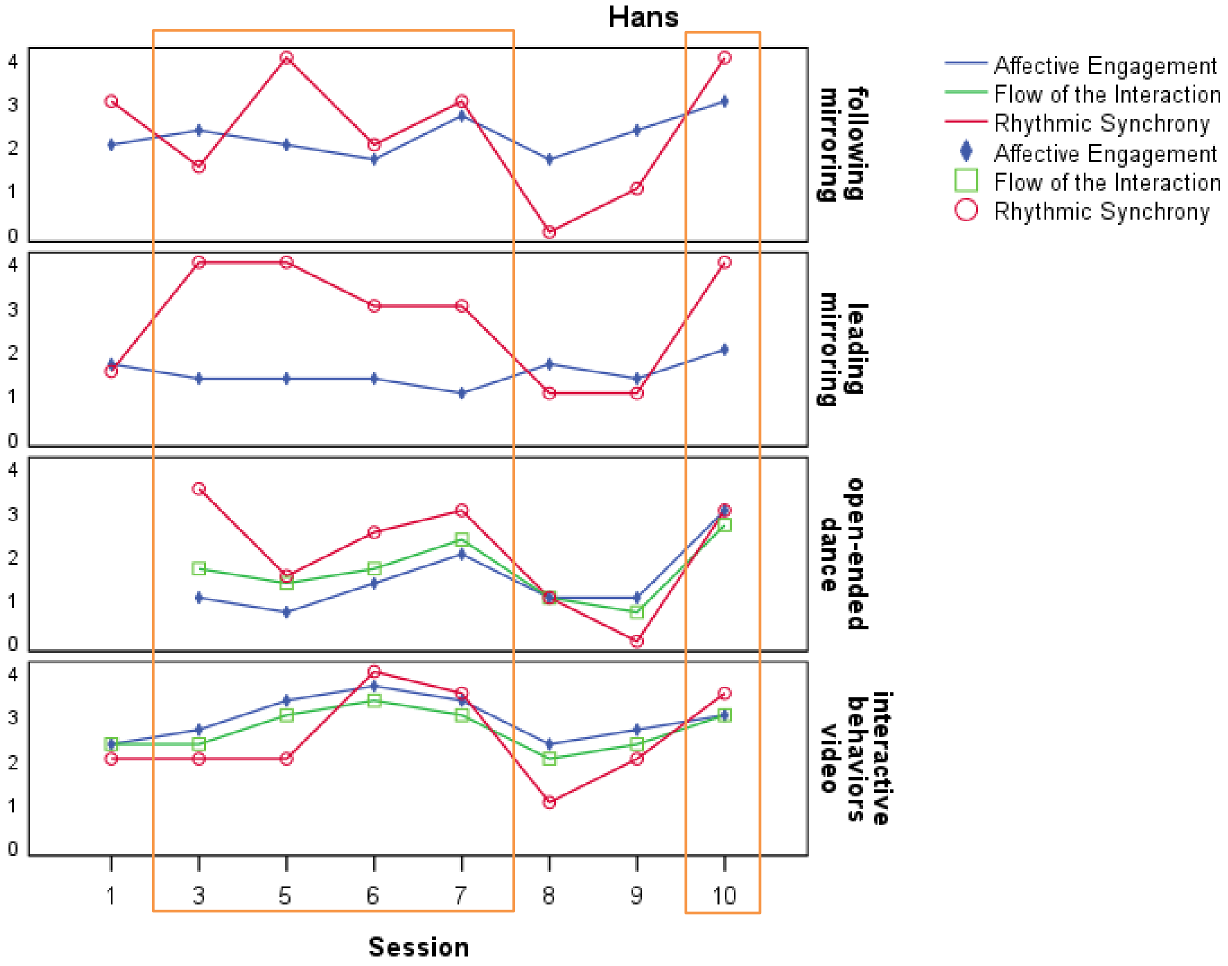


**Supplementary Figure 1.** Change over time by video type for Hans (pseudonym) showing affective engagement, flow of the interaction, and synchrony with his partners. Graphs separated by video segment type with change over time for the 30 second videos of leading mirroring, following mirroring, the open-ended dance, and one video selected for the most interactive behaviors displayed during one of these three tasks. Due to videographer error, video was missing for the open-ended dance in session one. Flow of the interaction was not scored in the following and leading video segments. Orange boxes indicate the sessions for which Hans had the same partner, showing an increase in affective engagement over the open-ended dances in the sessions with this repeated partner only.

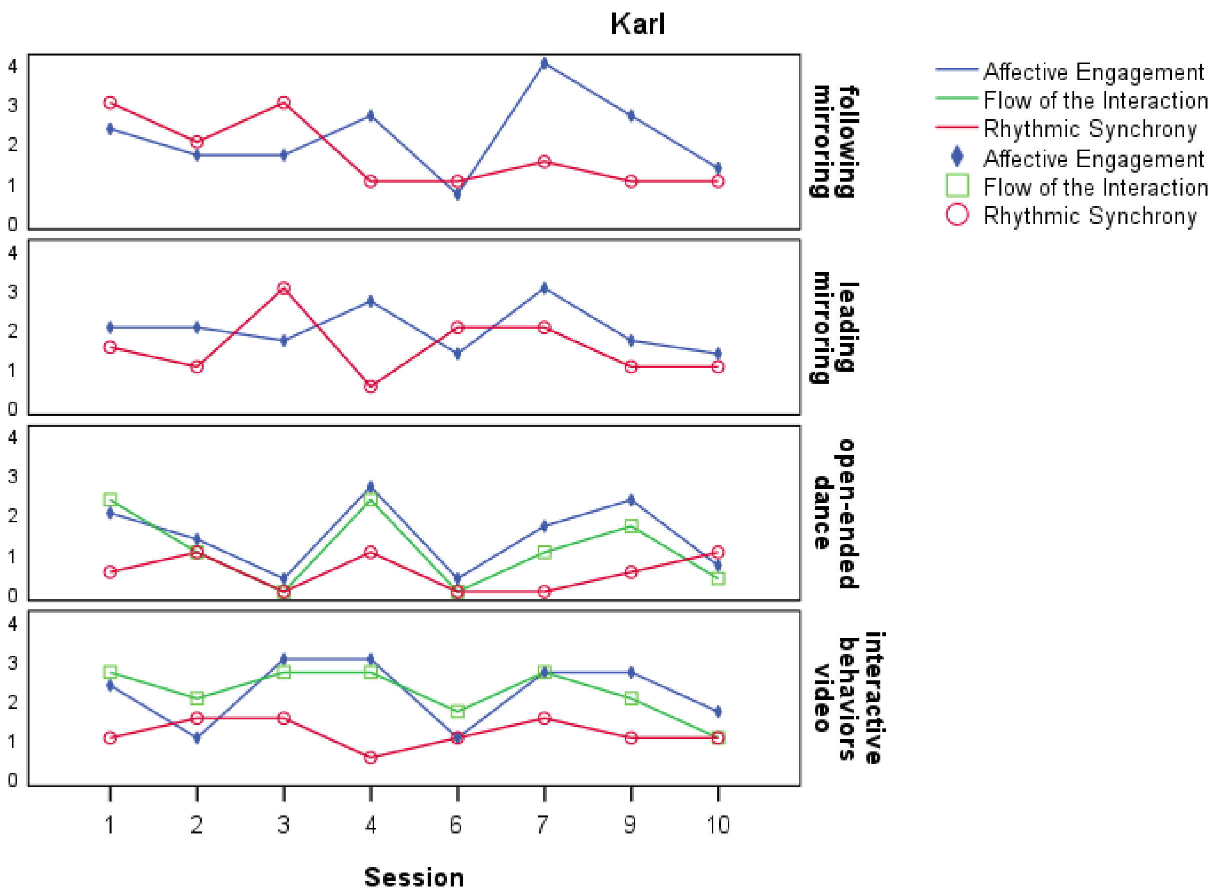


**Supplementary Figure 2.** Change over time by video type for Karl (pseudonym) showing affective engagement, flow of the interaction, and synchrony with his partners each week. Graphs separated by video segment type with change over time for the 30 second videos of leading mirroring, following mirroring, the open-ended dance, and one video selected for the most interactive behaviors displayed during one of these three tasks. Flow of the interaction was not scored in the following and leading video segments.

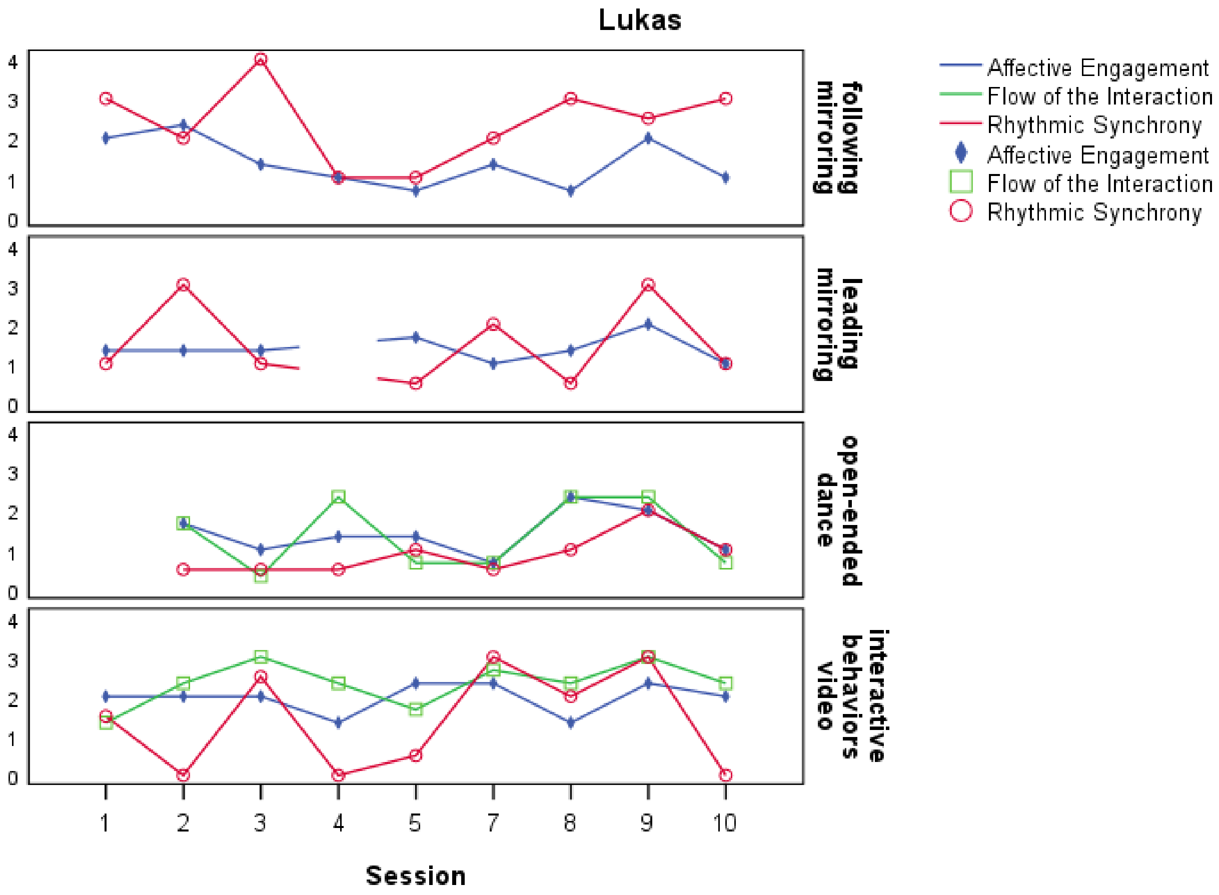


**Supplementary Figure 3.** Change over time by video type for Lukas (pseudonym) showing affective engagement, flow of the interaction, and synchrony with his partners each week. Graphs separated by video segment type with change over time for the 30 second videos of leading mirroring, following mirroring, the open-ended dance, and one video selected for the most interactive behaviors displayed during one of these three tasks. Flow of the interaction was not scored in the following and leading video segments. Due to videographer error, video did not show both Lukas and his partner during the open-ended dance in session one and leading mirroring in session four.

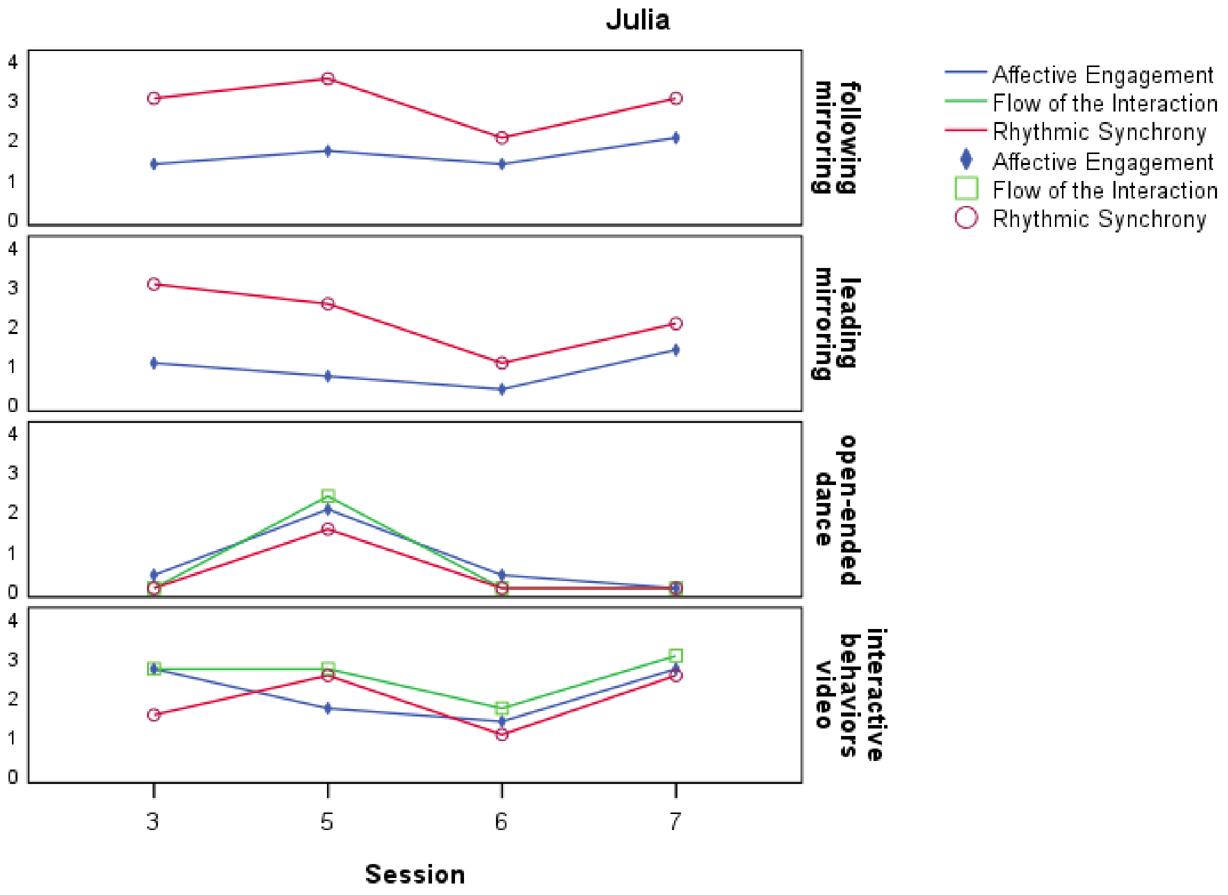


**Supplementary Figure 4.** Change over time by video type for Julia (pseudonym) showing affective engagement, flow of the interaction, and synchrony with her partners each week. Graphs separated by video segment type with change over time for the 30 second videos of leading mirroring, following mirroring, the open-ended dance, and one video selected for the most interactive behaviors displayed during one of these three tasks. Flow of the interaction was not scored in the following and leading video segments.

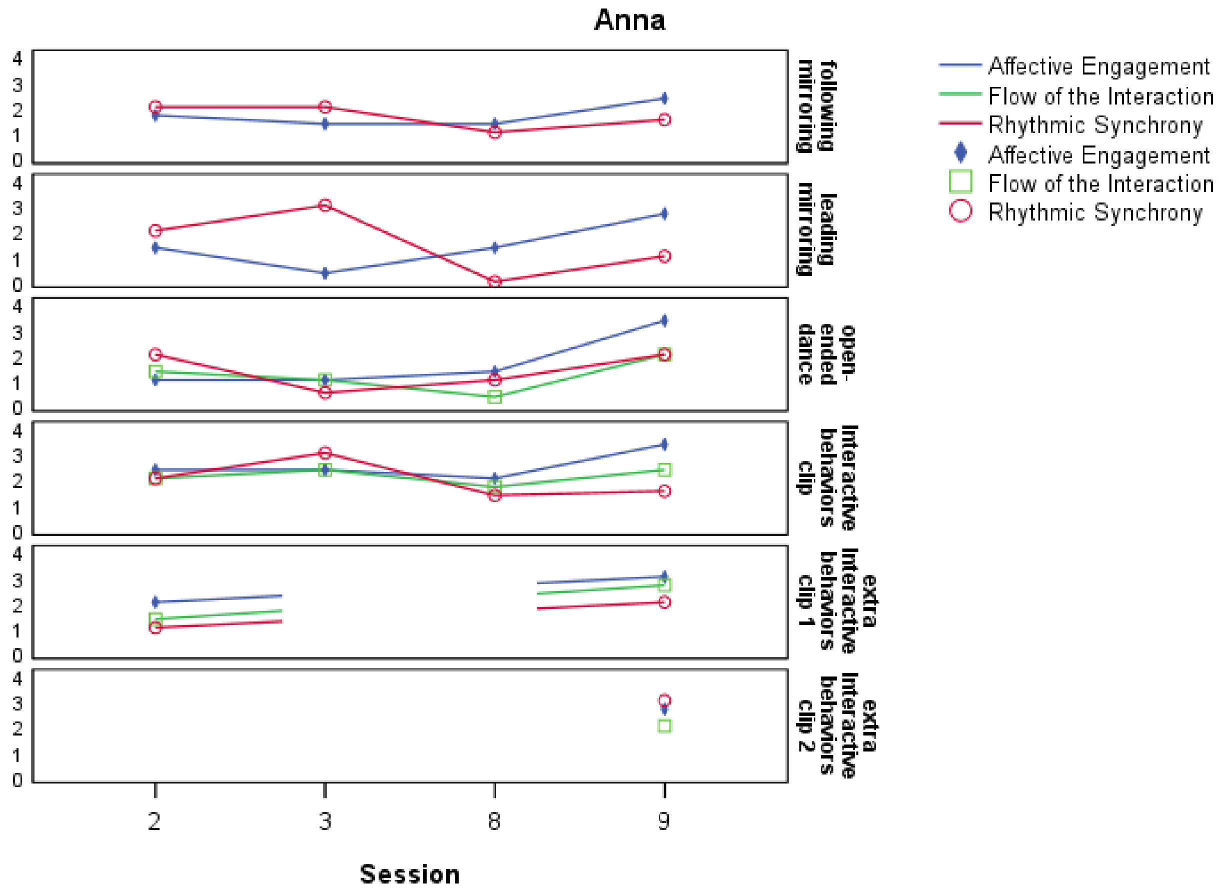


**Supplementary Figure 5.** Change over time by video type for Anna (pseudonym) showing affective engagement, flow of the interaction, and synchrony with her partners each week. Graphs separated by video segment type with change over time for the 30 second videos of leading mirroring, following mirroring, the open-ended dance, and one video selected for the most interactive behaviors displayed during one of these three tasks. Additional video segments showing interactive behaviors were selected in sessions with multiple interactive behaviors in a 30-second segment due to the low number of videos available for Anna. Flow of the interaction was not scored in the following and leading video segments.
